# What is the Best Way to Produce Consensus and Buy in to Guidelines for Rectal Cancer?

**DOI:** 10.1007/s11888-012-0121-x

**Published:** 2012-03-13

**Authors:** Rebecca K. S. Wong, James Brierley, Melissa Brouwers

**Affiliations:** 1 Princess Margaret Hospital, 610 University Avenue, Toronto, M5G 2M9 Canada; 2 Juravinski Hospital Site, G Wing, 2Fl, Rm 207, 711 Concession St, Hamilton, Ontario L8V 1C3 Canada

**Keywords:** Guidelines, Concordance, Rectal cancer, Radiotherapy, Ontario

## Abstract

Evidence-based guidelines are important tools and common pathways for translating evidence into clinical practice. It is most urgently needed when significant heterogeneity in practice exist. Actively engaging opinion leaders in the process of evidence-based guidelines development is important for several reasons. These include allowing the collective views of the practice communities to be represented, resolving heterogeneity in practice through discussion, and allowing credible recommendations to be formulated. Most importantly, the process itself is a tool for facilitating dissemination and implementation. Recognizing the gap between practice pattern and guideline recommendations, and devising strategies to address it represent an important step toward maximizing concordance between guideline and practice. Evidence-based recommendations serve as important reference points, against which we can measure, debate, and innovate from.

## Introduction

The management of curable rectal cancer has undergone significant change in recent years. Advances have been made in each of the disciplines involved in the care of patients with rectal cancer, with notable and significant improvements in surgery, radiotherapy, chemotherapy, radiology, and pathology. The strong interrelationship between these modalities means successful implementation of one strategy (or not) directly affects the readiness and appropriateness for change in another. For these reasons, strong, coordinated, and efficient process(es) are needed to optimize the uptake of evidence into practice, translating knowledge into improvement in patient outcomes.

Evidence-based guidelines are important tools, as well as a strategy for translating evidence into clinical practice. The reason why they are embraced by the medical community, is the methodological rigor available supporting the creation of evidence-based guidelines. Methods for evidence synthesis and appraisal have been well described and practiced [1]. Processes suitable for creating evidence-based guidelines for different medical communities have been established [2–5]. Tools to evaluate guideline quality such as the AGREE II instrument [6] are gaining international recognition and adoption. Databases to facilitate access and comparison between guidelines are comprehensive and accessible [7, 8]. Strategies for adapting guidelines from one jurisdiction to another, in an effort to share productivity and broaden generalizability, have also been described [9].

Despite these methodological advances, the final step between guidelines and adoption into clinical practice, i.e., implementation, remains an inexact science. While it would be ideal to have a template for efficient change implementation, a single set of strategy that would apply to all clinical domains or circumstances does not exist. Rather, general principles, which can be modified based on the clinical scenario, political environment, and organizational structure, are likely to provide the blueprint in moving forward.

What do we know about the best way of producing consensus and buy-in to evidence-based guidelines? In this paper, we will discuss what is known generally in this topic. Using the changing role of radiotherapy in the management of localized rectal cancer, we will describe how these principles are applied to motivate change and guideline implementation in a clinical practice environment.

## Strategies toward Guideline Consensus and Guideline Adoption: What Does the Literature Tell Us?

Multiple acceptable options, with weak evidence to guide selection between them translate into heterogeneity practice environments. The availability of multiple standards of practice makes it difficult to evaluate the quality of care, confusion for patients, and dilute the opportunity to refine excellence in selected strategies. Having clinical practice guidelines become most pressing when heterogeneity in treatment approach occurs within the practice environment.

The value of evidence-based guidelines lies far beyond the resulting recommendations alone. Rather, the process of guidelines development is itself a strategy by which buy-in and acceptance is nurtured and created. Guidelines development processes share a similar core structure. First, the process begins with the provision of a forum in which evidence is systematically brought together, appraised, and debated in order to reach consensus. This consensus is further reinforced and contextualized by external consultative and review processes. Second, the network of opinion leaders brought together for the task of building consensus and creating recommendations are often the same opinion leaders charged with dissemination and implementation at a local level. Similarly, these opinion leaders are often recruited into other networks, with the mandate and ability to initiate policy changes. It is this active engagement of “networks of opinion leaders” that is the cornerstone for dissemination and implementation inherent in many evidence-based guidelines development processes.

What does the literature tell us about the science of how to establish consensus among experts, and implementation strategies beyond the guideline development process itself? These questions are the subject of much research although the fields are still evolving.

### Consensus Methods

Even when high-quality evidence is available, our individual knowledge, interpretation of the evidence, and opinions are influenced by many factors including past experiences, peer opinions, time to learn, and critical appraisal skills to name just a few. These factors influence our recommendations and opinions even more when the available evidence is sparse. New evidence may encounter no barriers in “uptake” given its confirmatory nature to local practice, but in other contexts, be critiqued against a high threshold for acceptance when its conclusions go against standard practice. Consensus methods are called upon to narrow heterogeneity and allow the best collective opinion to be made.

Within the taxonomy used within consensus methods research, the most common method of establishing consensus in clinical practice is generally referred to as an “informal” process. Groups are brought together (e.g., tumor board), are free to interact with the aim of reaching an agreement. This could work well in many circumstances. Behavioral science research would highlight some disadvantages with this approach, however. Individuals often behave differently in the presence of peers. Some may feel pressured to alter their opinion especially if theirs are different from those of the group. Formal methods on the other hand are created with the basic assumption that by explicitly involving all members of a group, it is more likely that all the appropriate arguments will be considered and therefore more likely to lead to the “right” decision [10••]. It is generally more labor intensive. Three formal methods most frequently used in health care research and modified for guidelines consensus development are described below.

The Delphi method involves the use of questionnaires where respondents are asked to express their recommendations or choices they feel the group should consider. The results are aggregated and returned to the respondent so that his/her response is juxtaposed against the response emerging from the group. From here, the individual is invited to modify his/her response. This approach minimizes the potential of dominant characters on the final decision, employs statistics to incorporate participants’ views, but places less emphasis on direct information exchange between participants.

The nominal group technique starts with each participant recording their ideas independently and privately. The ideas are then listed in a round-robin format until no new ones are expressed. The ideas are then discussed in turn. Finally, a voting process takes place and the individual judgments are synthesized statistically to arrive at the group’s final judgment.

The consensus development conference method involves bringing a select group of individuals together with the objective of arriving at consensus about an issue. Evidence is presented in an open forum, and the panel deliberate before arriving at a decision.

Most clinical guidelines development groups have employed informal, or versions of the consensus development conference methods. This is because of the strong parallel between achieving consensus in clinical guidelines and clinical decision-making. The validity of the recommendations hinge heavily on the breathe of the consultative process, composition of the panel, in addition to the rigor of the evidence. The best method to use would depend on the questions that need to be addressed [11, 12].

### Guideline Dissemination and Implementation Strategies

Explicit guideline dissemination and implementation strategies should ideally be planned, for each recommendation that calls for a change in practice. Which method(s) to use depend on a number of factors including the clinical context, who and what we are trying to change, as well as resource availability. The Cochrane Effective Practice and Organization of Care (EPOC) group described a set of taxonomy to facilitate reporting and communication in this domain. Common strategies that have been studied include educational materials, educational meetings, local consensus processes, educational outreach visits, local opinion leaders, patient-mediated interventions, audit and feedback, reminders, marketing approaches, and mass media.

Several systematic reviews addressing the question “which of the dissemination and implementation strategies are most effective” have been published and they share similar conclusions [13••, 14, 15••]. Clear enunciation of the known barriers and facilitators the strategies are intended to address should be considered. While there are some suggestions that multifaceted strategies have a stronger effect, perhaps owing to the fact that they are more likely to address multiple barriers, it may not yield proportionally more benefit. Costs and benefits of different strategies need to be considered especially when limited resources are available. It may be more efficient to use a cheaper, more feasible, but less effective intervention, than a more expensive but potentially more effective method.

### Measuring Adherence

The final step to confirm buy in and impact of guidelines is to examine the level of concordance between practice and guideline recommendations. Selecting the parameter(s) for measuring compliance is not always straight forward. The National Quality Measures Clearinghouse recommended that the parameters selected should have three core characteristics. It should be supported by evidence, have sound measurement properties, and be available and accessible within the timeframe(s) of interest [16].

Once the metrics are selected, making an explicit judgment on what level of guideline concordance is optimal, and what key confounding factors are required for interpreting the compliance level, requires careful consideration [17]. For example, patient preferences may result in a treatment decision different from what was recommended. Comorbid conditions may result in reversal of the risk-benefit ratio [18]. Frailty (a term encompassing declining functional reserve, conferring vulnerability to disability, hospitalization, nursing facility admission, and death) beyond the impact of age and known medical conditions deserves consideration [19–21]. For new procedures to be practiced, adequacy of training, provision of resources, and accessibility need to be considered. Administrative data sources used to generate guideline concordance data may underestimate compliance and audits to estimate the level of discrepancy will facilitate interpretation [22].

Degree of adherence to guidelines, be it strong or weak, is seldom solely attributable to the strength or quality of the guidelines themselves. There are many systems that may influence or impede behavior aligning with the recommendations. These include the characteristics of the primary studies, training of the clinical team, and appropriate human and financial resources to name a few. Understanding the specific contribution of the guideline per se is often challenging. While concordance indicators can represent a convenient way to set bench marks, providing sufficient information to help users interpret and understand the data correctly is required.

## The Clinical Context: Rectal Cancer and the Role of Radiotherapy

The same body of evidence should be available to answer the same question conducted by any reviewer. However, the way it is interpreted and applied can vary both at the individual and community level. How radiotherapy is used in the management of rectal cancer is a good example. Its application has evolved quite differently in Europe versus North America [23•]. An understanding of the differences in practice environments is essential to consider the promoters and barriers toward concordance with standards and recommendations.

In North America, the NIH consensus guideline was powerful in establishing the long course (5 weeks) postoperative pelvic radiotherapy as the standard of practice in the 1990s [24]. Since then there has been a gradual shift toward adoption of preoperative long course chemoradiotherapy so that it became the dominant practice pattern since the 2000s. While this was motivated by the conclusions from the Swedish [25] and Kapeteijn’s [26] studies published in 1997 and 2001, respectively, this adoption of preoperative radiotherapy in North America was only in part consistent with the key evidence available at the time [25, 26]. Despite the fact that both studies employed the short-course (5 days) regimen, the use of the long course (5 weeks), a translation of the postoperative regimen into the preoperative setting which adheres to the use of standard dose per fraction (2 Gy) was generally used. The reasons are likely multifactorial. North American radiation oncologists subscribe to the radiobiological principles that standard dose per fraction is less likely to cause late toxicities and in the absence of evidence at the time, “believed” that the standard dose per fraction is at least equivalent (if not potentially superior) to the SCRT that was used within the primary studies. The transition of delivering a 5-week course of radiotherapy postoperatively to preoperatively has little resource implications and was readily implementable. There were also other forces at play. For example, preoperative radiotherapy was embraced by our surgical colleagues in part owing to the positive clinical observations of improved resectability and “minimal” incremental perioperative complications, facilitating rapid adoption. The use of SCRT on the other hand, requires rapid coordination between radiotherapy and surgery which present practical challenges.

The evolution in clinical practice in Europe was quite different. In Sweden, where short-course preop RT was first demonstrated to confer a survival benefit, SCRT was the dominant practice in 2001 [27] reflective of effective uptake of the results from the Swedish rectal trial [25], as was the practice in the UK [28•]. The publication of the UK MRC-led study CR07 NCIC CTG CO16 [29] in 2009 reinforced the superiority of short-course radiotherapy (SCRT) over selective postoperative radiotherapy [30].

The evidence as described in the EORTC study in support of the use of long-course CRT preoperatively came after in 2004 [31]. In North American, this evidence is easily adopted since it is consistent with the standard practice of using long-course CRT. Its interpretation within European practice required more active debate.

The primary evidence by Bujko et al. suggesting no difference between short-course and long-course chemoradiotherapy [32] was at once interpreted as consistent with ongoing practice of SCRT in Europe, and consistent with long-course radiotherapy in North America.

## From Consensus Development to Implementation: Ontario Rectal Cancer Guideline

The Ontario rectal cancer evidence-based guidelines were created under the auspices of the Cancer Care Ontario Program in Evidence-Based Care. The heterogeneity factor, with multiple acceptable approaches, has motivated each update to our guideline (Table 1). The first rectal cancer guideline published in 2000 [33] addressed whether preoperative radiotherapy should be used in patients with resectable rectal cancer. The recommendations supported the use of postoperative radiotherapy and/or chemotherapy for resected stage II or III disease, emphasizing the benefit of being able to add radiotherapy selectively only to patients who need it when the decision is made based on pathological stage. The second rectal cancer guideline published in 2003 [34] recommended preoperative radiotherapy as an acceptable alternative to postoperative treatments. The third guideline published in 2010 recommended the use of preoperative chemoradiotherapy (long course). Preoperative radiotherapy alone with long course, short course, or postoperative chemoradiotherapy were all considered acceptable standards in addition. Rationale guiding the choice between these options were deliberated [35•].

**Table 1:** Summary of key questions and recommendations for radiotherapy and rectal cancer from the Cancer Care Ontario Program in Evidence-Based Medicine

Question 2000 [33]	Should we use preoperative radiotherapy in patients with resectable rectal cancer to improve local recurrence and survival?
Recommendation	Randomized trials demonstrate that radiotherapy before surgery is significantly more effective than surgery alone in reducing local recurrence and probably death in patients with resectable rectal cancer.
	However, preoperative radiotherapy requires treatment of most rectal cancer patients regardless of the stage of the disease and consequent exposure to the risk of radiation-induced morbidity and mortality. Furthermore, when considering all rectal cancer patients, stage-selective postoperative radiotherapy is as effective as less stage-selective preoperative radiotherapy and should remain the standard treatment.
	Patients with evidence of advanced clinical stage but resectable rectal cancer should be encouraged to participate in clinical studies testing the role of preoperative radiotherapy alone or combined with chemotherapy.
Question 2003 [34]	Should patients with resectable rectal cancer receive preoperative radiotherapy to improve survival and local recurrence?
Recommendation	• Preoperative radiotherapy is an acceptable alternative to the previous practice of postoperative radiotherapy for patients with stage II and III resectable rectal cancer.
	• Both preoperative and postoperative radiotherapy decrease local recurrence but neither improves survival as much as postoperative radiotherapy combined with chemotherapy. Therefore, if preoperative radiotherapy is used, chemotherapy should be added postoperatively, at least to patients with stage III disease.
Question 2010 [35•]	Following appropriate preoperative staging tests, should patients with resectable stage II/III rectal cancer be offered preoperative radiotherapy (with or without chemotherapy)?
Recommendation	• Preoperative chemoradiotherapy is preferred, compared with preoperative radiotherapy (standard fractionation: longer course 45–50.4 Gy in 25–28 fractions) alone, to decrease local recurrence.
	• Preoperative chemoradiotherapy is preferred, compared with a postoperative approach, to decrease local recurrence and adverse effects.
	• For patients with relative contraindications to chemotherapy in the preoperative period, acceptable alternatives are preoperative standard fractionation (longer course 45–50.4 Gy in 25–28 fractions) or hypofractionation (short course 25 Gy in five fractions) radiotherapy alone followed by surgery, guided by the risk of adverse effects.
	• Patients eligible for preoperative radiotherapy with or without chemotherapy should also be considered for adjuvant chemotherapy.

### Consensus Development, Dissemination, and Implementation

Inherent within the guidelines development process, achieving consensus hinges first on the active engagement of a “network of opinion leaders.” Within the Program in Evidence-Based Care for Cancer Care Ontario, this began with the composition of the guidelines expert panel itself. Each expert panel consists of opinion leaders, representing the relevant disciplines, the geographic diversity across the province, and methodologists. The topic formulation and prioritization is driven by the group itself to represent the needs of the community it represents. The guideline development cycle [2] includes rigorous methodologies for question definition, evidence synthesis, and crafting of the recommendations. The recommendations are presented to the users accompanied by a summary of the key evidence and qualifying statements. These statements, while succinct, are crafted carefully, and reflect several iterations, modifications, and consensus achieved through discussion.

An example behind the most recent version of the rectal guideline perhaps serves to illustrate the effect of these consensus discussions. The final guideline recommended the use of radiotherapy for all patients with SII and III disease. Significant discussion occurred during the recommendation formulation as to whether there are subgroups of patients where the benefits of preoperative radiotherapy outweigh its toxicities such as in higher rectal lesions, node-negative disease, and in expert hands where high-quality TME procedures are anticipated. While the panel acknowledged that under these specific circumstances, the toxicity may outweigh the benefits, the appropriateness of articulating this within the guidelines were felt to be premature within our practice environment. The ability to incorporate these refinements within the guideline requires uniformity in reporting standards for staging MR and pathology, which was still in development across the province. The panel members acknowledged the variation in opinions but arrived at a consensus to recommend preoperative radiotherapy including these subgroups, recognizing that refinements could occur in time. A second consensus debate occurred around the use of SCPRT. This was not commonly considered across Ontario. The implementation of SCRT, while supported by evidence, represents a change from our standard practice. The rationale for change and circumstances when this should be used, relative to the standard approach was needed. The final recommendation wording of “hypofractionated (short course 25 Gy in five fractions) radiotherapy is an acceptable alternative to standard long course chemoradiotherapy” represents the panels’ adaptation of the evidence into our practice environment.

Prior to formal adoption and dissemination, the draft documents go through a series of reviews including an internal and external review with the ultimate goal of maximizing its quality and credibility. The internal review panel (2–3 reviewers) is charged with the task of ensuring each PEBC document is developed in a methodologically rigorous fashion and that the recommendations were supported by the evidence in a transparent way. The external review process included soliciting opinion from oncologists within Ontario and targeted external peer reviewers [36]. Review comments and changes to the original recommendations were synthesized and incorporated in the guidelines document itself.

Finally, dissemination was accomplished by posting of the guidelines onto the central website [37], registration in the Guidelines clearinghouse [8], evaluation and posting with the Inventory of Cancer Guidelines with the Canadian Partnership Against Cancer (http://www.cancerguidelines.ca; http://www.cancerview.ca/portal/server.pt/community/sage/521/sage), and publication of the systematic review and recommendations in peer-reviewed journal [35•].

The multiple-step process meant that our complete guideline development cycle typically takes approximately 18 months to complete. In clinical areas where there are active changes in clinical practice processes (e.g., availability of high-quality MRI for staging in clinical environment where this was not readily accessible previously) or where high-impact clinical trial results are released, matching response to a rapidly changing environment could be challenging. Mechanism is in place to ensure available guidelines were last updated within 5 years, however.

While designing an explicit implementation plan was beyond the scope of the Program in Evidence-Based Care, the active engagement of the network of opinion leaders continues to exert its influence in promoting clinical uptake and adoption beyond the dissemination stage. At a physician level, Ontario oncologists uniformly participate in regular multidisciplinary tumor boards serving as a forum for real-time peer review. The opinion leaders serve as advocates for the provincial recommendations in these settings. The guideline recommendations became current topics and agenda items at regional conferences allowing further deliberation and alignment of practices to occur. The opinion leader network charged with creating these recommendations overlaps, and works synergistically with networks charged with creating recommendations in other related topics such as imaging [38], surgery and pathology [39], and multidisciplinary case conferences [40]. At a policy maker level, Cancer Care Ontario, the formal cancer program for the province of Ontario (of which the Program in Evidence-Based Care is a part of), has a breadth of levers to facilitate the application of evidence into practice and policy formation. For example, the Cancer System Quality office, charged with the mandate of reporting on the state of the Ontario cancer system, incorporates guideline adherence parameters as part of our “report card” on cancer care. Educational medical workshops and performance management strategies form part of the response to published quality metrics. High-quality evidence-based guidelines developed within the PEBC set off a ripple effect that is central to facilitating buy in, implementation, and steps for future improvements.

### How Concordant Are We?

The percentage of patients with SII and III rectal cancer receiving CRT were felt to be the most useful quality measures for the guidelines relevant to our rectal guidelines [24]. This is supported by several systematic reviews [41, 42]. The availability of this parameter was limited by the reporting of stage, and details of radiotherapy prescription at a provincial level, and requires some degree of inference. The Cancer Quality Council of Ontario (CQCO) reported on the practice pattern for rectal cancer in 2002, suggesting the proportion of patients treated with preoperative radiotherapy was approximately 15%. The Canadian Partnership Against Cancer provided a quality report using 2007 data reporting the proportion of patients receiving any radiotherapy was 52% in Ontario while the proportion receiving this preoperatively was 50%. In 2009 Ontario data would suggest while the proportion of patients receiving any radiotherapy remains stable at approximately 50%, the proportion receiving this preoperatively has risen to 62% (Unpublished data). These practice patterns align with the recommendations in 2000 for postoperative CRT, 2003 for preoperative RT as an acceptable alternative, and 2010 for preoperative CRT as the standard of practice.

These findings cannot support a cause and effect between our guidelines and practice pattern. What is clear, however, is that the rigorous process of developing high-quality evidence-based guidelines created by a network of opinion leaders allows differences in opinion between opinion leaders to be explicitly discussed, consensus reached (or at least differences articulated), and what is needed to further advance patient outcomes described. The convergence of opinions through this process meant the availability of transparent sets of recommendations and benchmarks (and the rationale behind them) for the communities, its practitioners, and patients. Evidence-based guideline recommendations serve as important references against which we measure, debate, and innovate from.

## Conclusions

Evidence guiding the best consensus building, guidelines dissemination, and implementation strategies is incomplete but growing. A network of opinion leaders built for creating evidence-based guidelines, actively engaged by an effective consensus development process, represents one of the most effective ways toward achieving excellence in quality of care. Evidence-based recommendations serve as important reference points, against which we can measure, debate, and innovate from. 
